# Evaluating co-occurrence of depression and sexual dysfunction and related factors among Iranian rural women: A population-based study

**DOI:** 10.37796/2211-8039.1003

**Published:** 2020-03-28

**Authors:** Raheleh Khademi, Seyed Hamzeh Hosseini, Hamid Sharif Nia, Soghra Khani

**Affiliations:** a Student Research Committee, School of Nursing and Midwifery, Mazandaran University of Medical Sciences Sari, Iran; b Department of Psychiatry, Psychiatry and Behavioral Sciences Research Center, School of Medicine, Mazandaran University of Medical Sciences, Sari, Iran; c Department of Nursing, School of Nursing and Midwifery Amol, Mazandaran University of Medical Sciences, Sari, Iran; d Sexual and Reproductive Health Research Centre, School of Nursing and Midwifery, Mazandaran University of Medical Sciences, Sari, Iran

**Keywords:** depression, physiological, sexual dysfunction, sexual health, women

## Abstract

**Background and objectives:**

Sexual dysfunction and mood disorders have a high prevalence rate and their co-occurrence has been reported in previous studies. This study aimed to determine the prevalence of co-occurrence of sexual dysfunction and depression and related factors in women.

**Materials and methods:**

This descriptive-analytical study was carried out on 826 married rural women aged 15-49 years in Sari, Iran in 2018, selected by random sampling. The participants filled the demographic and fertility questionnaires, as well as Beck's Depression Inventory and Female Sexual Function Index (FSFI).

**Results:**

In this study, 18% of the participants experienced the co-occurrence of depression and sexual dysfunction. In addition, results of the multiple logistic regression showed that forced marriage (OR = 0.31, CI 95%: 0.15 to 0.64, *P* < 0.001), a one-level increase in the education of the spouse (OR = 0.76, CI 95%: 0.59 to 0.98, *P* < 0.041), lack of history of depression (OR = 0.36, CI 95%: 0.20 to 0.66, *P* < 0.001) and lack of vaginal infection (OR = 0.41, CI 95%: 0.27 to 0.62, *P* < 0.001) were considered as factors contributing to a decline in the co-occurrence of depression and sexual dysfunction. On the other hand, not having a private bedroom (OR = 1.63, CI 95%: 1.09 to 2.43, *P* < 0.017), no vehicle (OR = 1.52, CI 95%: 1.02 to 2.27, *P* < 0.038), a history of sychiatric diseases (OR = 2.09, CI 95%: 1.2.0 to 3.65, *P* < 0.009), lack of chronic diseases (OR = 2.11, CI 95%: 1.03 to 4.31, *P* = 0.039) and lack of use of antidepressants (OR = 2.03, CI 95%: 2.03 to 1.03, *P* < 0.039) increased the co-occurrence of depression and sexual dysfunction.

**Conclusion:**

According to the results of the study, about one-fifth of the married rural women experienced the co-occurrence of depression and sexual dysfunction. If healthcare providers detect one of the disorders of depression or sexual dysfunction in a patient, it is suggested that the person be assessed in terms of the other disorder and the proper treatment be applied. Furthermore, the healthcare personnel must pay attention to factors related to the co-occurrence of these disorders in addition to providing a treatment program.

## 1. Introduction

The World Health Organization (WHO) de-fines sexual dysfunction as “the various ways in which an individual is unable to participate in a sexual relationship as he or she would wish” [1]. Generally, disorders of desire, arousal, orgasm, and sexual pain disorders are recognized as sexual dysfunction. These issues are caused by anatomical, physiological, medical and psychological factors that might cause severe discomfort in individuals and adversely affect their quality of life and interpersonal communications [2]. Various levels of frequency of female sexual dysfunction (19.2–89%) have been reported in the Iranian population [3,4]. One of the reasons for the high prevalence of sexual dysfunction in Iranian women is the cultural and social barriers, as well as taboos, misconceptions and also insufficient education about sexuality before marriage [5,6]. While a global study revealed that there is at least one sexual dysfunction in 41% of women [7] in a systematic and meta-analysis study among reproductive-age women in Iran indicate that the pooled prevalence rate of sexual dysfunction was estimated as 52% [8]. Sexual issues affect the body, mind and social behaviors of individuals and jeopardized sexual behaviors cause problems in emotions related to sexual function, character, as well as social, emotional and friendship functions. Studies have shown that dissatisfaction with sexual function is closely related to social issues, such as crimes, sexual assaults or psychological diseases [9]. Moreover, sexual dysfunction can lead to separation, divorce, and depression [10]. Generally, sexual function and desires are an important part of women's health [11]. Also, healthy female sexual function plays an important role in health and improved quality of life [12–14]. Factors affecting sexual response including the general health status, psychological disorders, chronic diseases [15], social issues [16], aging, drugs, pelvic surgery, benign or malignant gynecologic diseases, menstrual cycle, pregnancy, breastfeeding and infertility [17,18], childhood incidents, former sexual experiences and economic status [19].

It has been previously shown that severe depression is common in women of reproductive age [20]. The childbearing period and years of child-rearing are a peak time of vulnerability to depression in women. In addition, depression starts at younger ages (since 30 years old) and declines after the age of 45 years. There is no evidence to show that depression increases during menopause [21,22]. Studies have shown that changes in emotional symptoms and sexual responses occur concomitantly [23]. In fact, both sexual dysfunctions and mood disorders have a high incidence rate and there is a high co-occurrence between them [24]. A high rate of women diagnosed with mild to severe depression has experienced sexual dysfunction [25,26].

In Iran, about one-third of the population of women (near 37 million) are rural women [27], who are a vulnerable group [28,29]. In rural societies, women marry at young ages and belong to low-income families. In addition, they have no jobs and a low level of education [30]. On the other hand, cultural beliefs have a profound effect on normal sexual function and can lead to sexual dysfunction. In rural societies, the existing culture considerably affects the beliefs and attitudes of individuals. Putting pressure on individuals to have a child early in marriage causes anxiety and sexual dysfunction, turning marital relations into duty and not a desirable act, which leads to reduced sexual desire [31]. Furthermore, sexual dysfunction is more prevalent in rural women, compared to urban women [27].

Isolation of rural women along with expectations of traditional roles, economic pressures and lack of support result in their depression and increase their vulnerability [32]. The results of studies have shown that changes in symptoms of depression are concomitantly associated with sexual function fluctuations on a daily basis [23]. In addition, sexual dysfunction is more reported in depressed women [33]. In general, women living in rural areas experience more distress or turmoil, compared to women inhabiting urban areas, and cultural issues in villages limit the acceptance of mental health services in these regions [34]. In a research in one of the villages of India, one-seventh of the rural women suffered from more than one type of sexual dysfunction [35]. Similar to physical and psychological problems, sexual disorders should be identified and effective measures should be taken to eliminate them. Today, less attention is paid to the role of psychological factors in relation to the biological factors of these disorders [36].

To date, no comprehensive study has been performed on the prevalence of depression and sexual dysfunction among women in rural area. This study aimed to determine the incidence of co-occurrence of depression and sexual dysfunction and related factors among rural women.

## 2. Methods

This population-based cross-sectional study was performed on married women aged 15–49 years, who referred to the comprehensive rural healthcare centers of Sari in 2018 and met the inclusion criteria. Sample size was determined at 826 using G-Power 3.1.7 software based on the previous study [10].

At first, the city of Sari was divided into four section. Afterwards, the number of eligible women in each comprehensive rural healthcare center was extracted from the health unit of Sari, and the share of each section and each center in rural areas determined (Table 1).

By using a random number table, participants selected and contacted by the researcher to receive the necessary explanations. If intending to participate in the study, the selected women were encouraged to refer to the healthcare centers to fill the questionnaire. After referral, the married women anonymously filled the questionnaires. Study tools included demographic and individual characteristics questionnaire, the standard Beck's Depression Inventory, and the Female Sexual Function Index (FSFI) to assess sexual dysfunction of the participants. The validity and reliability of these questionnaires have been assessed in many studies and were known around the world [2,20,26].

Inclusion criteria were the age range of 15–49 years, being married, being covered by comprehensive rural healthcare centers, having at least one child, having the minimum level of education (the ability of reading and writing), and lack of menopause, infertility, pregnancy, and breastfeeding. Exclusion criteria were experience of crisis in the past six months (death of loved ones, losing a job, or retirement), addiction to alcohol and drugs, and living separately from husband for any reason.

At first, we identified women with sexual dysfunction using the FSFI. Scores of 28, 3.3, 3.4, 3.4, 3.4, 3.8, and 3.8 were considered as cut off points for variables of total scale, desire, mental stimulation, lubricating, orgasm, satisfaction, and pain, respectively. In other words, scores greater than the cutoff point, indicated a good sexual function [2,26]. We simultaneously identified women with depression using Beck's Depression Inventory (women were divided into two categories of lack of depression or mild depression with a cutoff point of <20 and moderate-severe depression with a cutoff point ≥20) [20].

## 3. Statistical analysis

Data analysis was performed by SPSS 25 using descriptive statistics to describe the qualitative variables (marital status, level of education, occupational status, having a private bedroom, underlying diseases, diagnosed psychological diseases, taking antidepressants, using narcotics, co-occurrence of sexual dysfunction and depression, marital satisfaction, economic status, sufficient family income, consuming alcohol, menopause, infertility, pregnancy, and breastfeeding) and mean (standard deviation) to describe the quantitative variables (age, age at marriage, age of spouse, duration of marriage, number of children, history of former marriage and forced marriage) applied (χ2) used to determine the relationship between depression and sexual dysfunction. In addition, a multiple logistic regression model was applied to determine the factors related to the co-occurrence of depression and sexual dysfunction. Women with both sexual dysfunction and depression were selected, followed by the formation of a new dichotomous variable (co-morbidity). Women selected by this way were allocated the code 1 and the rest of the participants were assigned the code zero. Next, the significant variables (Table 2) that were estimated by chi square (χ2) were entered in to the multiple logistic regression model. The model used for the entrance of variables into the model was the standard model (enter). *P*-values less than 0.05 were considered statistically significant.

## 4. Results

In total, 826 women from 34 healthcare centers of four section of the city were recruited and evaluated in this study. This study the non-response rate was zero and all participants returned the questionnaire. In this study, 5.4% (n = 45) of women reported forced marriage, and the highest level of education of their spouses was a junior high school degree (48%; n = 397) and 68.8% (n = 569) of women had private bedrooms. Moreover, 11.7% (n = 97) of women had a history of depression. Regarding the prevalence of chronic diseases, 4.7% (n = 39), 5.4% (n = 44), 3% (n = 25), 12.5% (n = 104), 4.8% (n = 40), and 6% (n = 50) of women had diabetes, hypertension, cardiovascular diseases, thyroid diseases, neurological diseases, and other chronic diseases, respectively. However, 63.4% (n = 524) of the participants had no disease. In this respect, the most prevalent chronic disease among the participants was thyroid disorders. In terms of evaluation of the use of antidepressants, 2.3% (n = 18), 3.8% (n = 32), and 3.1% (n = 26) of the participants used fluoxetine, alprazolam, and other antidepressants (amitriptyline, citalopram, sertraline, imipramine, doxepin, and nortriptyline), respectively. On the other hand, 90.8% (n = 750) of the participants used no antidepressants, 25.3% (n = 209) of women had vaginal infections, 64.8% (n = 536) of women had vehicle, 5.8% (n = 48) of their wives had previous marriage, the highest level of education of women was diploma and higher degree (67.9%; n = 562), 72.1% (n = 596) of women were housewives, and 67.9% (n = 561) of women had menstrual interval 26-30 day. In terms of mental illness, 10.4% (n = 86), 4.9% (n = 41), 0.1% (n = 1) and 0.1% (n = 1) of women had anxiety, depression, schizophrenia and hallucination and 1% (n = 5) women had history of child abuse (Table 2).

In this study, the co-occurrence of depression and sexual dysfunction in rural women of Sari was reported at 18% (CI 95%: 15.38%–20.62%). 98.7% of depressed women had sexual dysfunction and 18.5% of women with sexual dysfunction had depression. There was a significant relationship between forced marriage, level of education of spouse, having a private bedroom, a history of depression, using antidepressants, and vaginal infection with the co-occurrence of depression and sexual dysfunction. The results of the multiple logistic regression showed that in individuals with a history of forced marriage (OR = 0.31), a one-level increase in education of spouse (OR = 0.76), lack of history of depression (OR = 0.36), and lack of vaginal infection (OR = 0.41) were regarded as supportive factors that reduced the co-occurrence of depression and sexual dysfunction. On the other hand, no separate bedroom (OR = 1.6), a history of psychiatric diseases (OR = 2), no history of chronic diseases compared to diabetes and (OR = 2.1), no having vehicle (OR = 1.52) and no use of antidepressants (OR = 2.0) increased the co-occurrence of depression and sexual dysfunction (Table 3).

## 5. Discussion

According to the results of the present study, the co-occurrence of depression and sexual dysfunction was 18% in rural women in Sari. To date, no study has been performed on rural women, especially in Iran. Aiming at assessing the relationship between sexual dysfunction and depression levels in women of Shiraz, Parpaei et al. concluded that 19.4% of the patients with mild depression had sexual dysfunction. In addition, the participants with severe depression had 14.1% sexual dysfunction [37], which is in line with our findings.

In a study by Casper et al., performed to evaluate the physical symptoms of early emotional disorders and their relationship with depression, 72% of patients with depression had a loss of sexual desire [38]. In the largest study (ELIXIR) on sexual dysfunction in 4557 depressed patients, 52.8% of untreated patients and 54.7% of patients taking antidepressants reported a decrease in sexual desire. Moreover, 76% of untreated patients reported arousal problems, whereas 37.9% of the participants had orgasmic dysfunction [39]. Angst et al. assessed the sexual problems in healthy and depressed participants, reporting loss of sexual desire, sexual dysfunction, and increased sexual desire in 35%, 26%, and 9% of women, respectively. The prevalence of sexual problems in depressed people was twice that of non-depressed people. Also, some types of sexual problem were found in 26% of normal subjects [40]. Assessing the effect of sexual dysfunction on depression, Mathew et al. reported that 31% of the participants had a loss of sexual desire and 34% had orgasmic dysfunction [41].

The difference in the prevalence of sexual dysfunction among women in different studies-might be due to the different population and the research tools. In some countries, such as India, sexual issues are taboo and there are sexual misconceptions in this country, and each country's culture has an impact on the prevalence of sexual dysfunction [42]. In addition, the prevalence of female sexual dysfunction might be reported lower than its actual rate due to embarrassment and shame of women [37].

According to the results of the present study, a significant relationship was observed between the co-occurrence of depression and sexual dysfunction and variables of forced marriage, level of education of spouse. Nonetheless, no relevant study was found in this regard. In a study has been shown that there was no association between the level of education of spouse and sexual dysfunction [43]. On the other hand, there was a significant relationship between the co-occurrence of depression and sexual dysfunction and having a private bedroom. The same relationship was observed in another study [10]. Our findings demonstrated a significant relationship between depression and sexual dysfunction of women, which has been reported by several studies [10,23,37,39,42,44].

In a review study, women with higher mental health had better sexual satisfaction [45]. The use of antidepressants was another major factor in this regard. Finding this association, Clayton marked that bupropion and nefazodone had the least effect on sexual dysfunction while SSRI and antidepressants had the greatest impact on this variable [46]. Vaginal infection was another factor contributing to the co-occurrence of depression and sexual dysfunction, it was significantly correlated with female sexual dysfunction [47]. This congruent in these studies might be due to pain or vaginal odorous discharges which.

According to the results of the present research, one of the factors relating the co-occurrence of depression and sexual dysfunction were chronic diseases (diabetes, hypertension, cardiovascular, thyroid, and neurological disorders). In a study by Safarnejad et al., chronic diseases were associated with female sexual dysfunction [48], which is in congruence with the results obtained by Cayan et al. [49]. Previous studies reported the existence of sexual dysfunction in patients with a history of depression, with associated atherosclerosis, hypertension, and diabetes [50,51].

In an American study, cardiovascular diseases, diabetes, depression, and urinary system disorders were identified as factors associated with sexual dysfunction [52]. Nevertheless, the history of physical diseases had no impact on sexual disorders [53]. Similarly, no such association was observed in the study by Hisasue et al. [54]. These differences might be due to sample size, inclusion criteria, and lack of report by patients.

This study has some limitation. Due to educational status of the study population, one of the main limitations of this study was using self-administered questionnaires for data collection. Also, honesty of the participants to respond to the questionnaire is important issue and therefore the accuracy cannot be determined. Furthermore, in questionnaire based surveys, recall bias is a general limitation. Additionally, due to the cultural background of study participants, answering to questions regarding their sexual activity was often difficult, which may influence of the results of the study.

## 6. Conclusion

According to the results of the current research, about one-fifth of the married rural women of Sari experienced the co-occurrence of depression and sexual dysfunction. There was a significant relationship between forced marriage, level of education of spouse having a private bedroom, a history of depression, using antidepressants, and vaginal infection with the co-occurrence of depression and sexual dysfunction. Women's health care providers should pay more attention to women's sexual health and the relevant factors (e.g., depression) and provide counseling services. Timely treatment is a preventive measure of sexual dysfunction in depressed women.

## Figures and Tables

**Table 1:** Description of study setting.

Sections of the city	Numbers of healthcare centers in the section	Numbers of married women aged 15–49 years	Sample allocation of each healthcare centers
North section	7	7855	189
South section	15	9340	224
East section	6	8248	198
West section	6	8946	215

**Table 2:** Socio-demographic characteristics of participants (N = 826).

Demographic characteristics		N (%)	Co-occurrence of depression and sexual dysfunction		*P*-value
			Yes	No	
Forced marriage	Yes	45 (5.5)	18 (12.1)	27 (4)	0.001
	No	781 (94.5)	131 (87.9)	650 (96)	
Level of education of spouse	Illiterate	33 (3.9)	11 (7.4)	22 (3.2)	0.002
	Elementary	246 (29.8)	52 (34.9)	194 (28.7)	
	Junior high school	397 (48)	72 (48.3)	325 (48)	
	Diploma and higher	150 (18.3)	14 (9.4)	136 (20.1)	
Private bedroom	Yes	569 (68.8)	87 (58.4)	482 (71.2)	0.002
	No	257 (31.2)	62 (41.6)	195 (28.8)	
	Yes	97 (11.7)	38 (25.5)	59 (8.7)	0.001
Depression-(diagnosed by a physicians)	No	729 (88.3)	111 (74.5)	618 (91.3)	
	Diabetes	39 (4.7)	5 (3.4)	34 (5)	
	Hypertension	44 (5.4)	10 (6.7)	34 (5)	
	Cardio vascular	25 (3)	5 (3.4)	20 (3)	
Underlying diseases	Thyroid	104 (12.5)	26 (17.4)	78 (11.5)	0.006
	Neurological	40 (4.8)	13 (8.7)	27 (4)	
	Other	50 (6)	14 (9.4)	36 (5.3)	
	None	524 (63.6)	76 (51)	447 (66.1)	
	Fluoxetine	18 (2.3)	7 (4.7)	11 (1.6)	
Antidepressants	Alprazolam	32 (3.8)	13 (8.7)	19 (2.8)	0.004
	Other drugs	26 (3.1)	10 (6.7)	16 (2.3)	
	None	750 (90.8)	119 (79.9)	631 (93.2)	
Vaginal infections	Yes	209 (25.4)	65 (43.6)	144 (21.3)	0.001
	No	617 (74.6)	84 (56.4)	533 (78.7)	
Have vehicle	Yes	536 (64.8)	83 (55.7)	453 (66.9)	0.009
	No	290 (35.2)	66 (44.3)	224 (33.1)	
	Housewives	596 (72.1)	108 (72.4)	488 (72)	0.001
Women's Job	Employed	161 (19.4)	19 (12.8)	142 (2 1)	
	Other	69 (8.5)	22 (14.8)	47 (7)	
Menstrual interval	20–25 Day	216 (26.1)	44 (29.7)	172 (25.4)	
	26-30-Day	561 (67.9)	94 (63)	467 (68.9)	
	31–35 Day	39 (4.7)	10 (6.7)	29 (4.2)	0.003
	>35Day	10 (1.3)	1 (0.6)	9 (1.5)	
Mental illness	Anxiety	86 (10.4)	38 (25.5)	48 (7.1)	
	Depression	41 (4.9)	20 (13.4)	21 (3.1)	
	Schizophrenia	1 (0.1)	0	1 (0.1)	
	Hallucination	1 (0.1)	1 (0.7)	0	0.001
	Other	0	0	0	
	None	697 (84.5)	90 (60.4)	607 (89.7)	
History of child abuse	Yes	5 (1)	3 (2)	2 (0.3)	
	No	821 (99)	146 (98)	675 (99.7)	
Previous marriage in women's wives	Yes	48 (5.8)	14 (9.4)	34 (5)	0.034
	No	778 (94.2)	135 (90.6)	643 (95)	
	Illiterate	5 (0.8)	3 (2)	2 (0.3)	
Level of education	Elementary	68 (8.2)	13 (8.7)	55 (8.l)	0.005
	Junior high school	191 (23.1)	35 (23.5)	156 (23)	
	Diploma and higher	562 (67.9)	98-(65.8)	464-(68.6)	

**Table 3:** Predicting factors for the co-occurrence of depression and sexual dysfunction in women: a multiple logistic regression.

Variable	OR	*P*-value	CI 95%
Forced marriage (yes/no)	0.31	<0.001	0.15–0.64
Level of education of spouse	0.76	<0.041	0.59–0.98
Vehicle (yes/no)	1.52	<0.038	1.02–2.27
A separate bedroom (yes/no)	1.63	<0.017	1.09–2.43
History of depression (yes/no)	0.36	<0.001	0.20–0.66
Diabetes	1	–	-
Hypertension (yes/no)	0.73	<0.55	0.26–2.02
Cardiovascular (yes/no)	1.20	<0.66	0.51–2.83
Thyroid (yes/no)	0.83	<0.75	0.27–2.57
Psychiatric (yes/no)	2.09	<0.009	1.20–3.65
No chronic diseases (yes/no)	2.11	<0.039	1.03–4.31
Using antidepressants (yes/no)	2.03	<0.039	2.03–1.03
Vaginal infection (yes/no)	0.41	<0.001	0.27–0.62
